# Identification of Germline Variants in Patients with Hereditary Cancer Syndromes in Northeast Mexico

**DOI:** 10.3390/genes14020341

**Published:** 2023-01-28

**Authors:** Diana Cristina Pérez-Ibave, María Lourdes Garza-Rodríguez, María Fernanda Noriega-Iriondo, Sonia María Flores-Moreno, Manuel Ismael González-Geroniz, Absalon Espinoza-Velazco, Ana Lilia Castruita-Ávila, Fernando Alcorta-Núñez, Omar Alejandro Zayas-Villanueva, Juan Francisco González-Guerrero, Adelina Alcorta-Garza, Oscar Vidal-Gutiérrez, Carlos Horacio Burciaga-Flores

**Affiliations:** 1 Servicio de Oncología, Centro Universitario Contra el Cáncer (CUCC), Hospital Universitario “Dr. José Eleuterio González”, Universidad Autónoma de Nuevo León, Monterrey 66451, Nuevo León, Mexico; 2 Hospital Regional de Alta Especialidad Materno-Infantil, Guadalupe 67140, Nuevo León, Mexico; 3 Instituto de Seguridad y Servicios Sociales para los Trabajadores del Estado (ISSSTE), Hospital Regional de Monterrey, Monterrey 64380, Nuevo León, Mexico; 4 Centro Médico Nacional Noreste, Instituto Mexicano del Seguro Social (IMSS), Unidad Médica de Alta Especialidad (UMAE) No. 25, Monterrey 64180, Nuevo León, Mexico; 5 ONCARE Treatment Center, Valle Unit, San Pedro Garza Garcia 66220, Nuevo Leon, Mexico; 6 Instituto Mexicano del Seguro Social (IMSS), Unidad Médica de Alta Especialidad, Hospital de Gineco Obstetricia (HGO) No. 23, Monterrey 64000, Nuevo León, Mexico

**Keywords:** pathogenic variants, hereditary cancer syndromes, genetic counseling

## Abstract

Hereditary cancer syndromes (HCS) are genetic diseases with an increased risk of developing cancer. This research describes the implementation of a cancer prevention model, genetic counseling, and germline variants testing in an oncologic center in Mexico. A total of 315 patients received genetic counseling, genetic testing was offered, and 205 individuals were tested for HCS. In 6 years, 131 (63.90%) probands and 74 (36.09%) relatives were tested. Among the probands, we found that 85 (63.9%) had at least one germline variant. We identified founder mutations in *BRCA1* and a novel variant in *APC* that led to the creation of an in-house detection process for the whole family. The most frequent syndrome was hereditary breast and ovarian cancer syndrome (HBOC) (41 cases with *BRCA1* germline variants in most of the cases), followed by eight cases of hereditary non-polyposic cancer syndrome (HNPCC or Lynch syndrome) (with *MLH1* as the primarily responsible gene), and other high cancer risk syndromes. Genetic counseling in HCS is still a global challenge. Multigene panels are an essential tool to detect the variants frequency. Our program has a high detection rate of probands with HCS and pathogenic variants (40%), compared with other reports that detect 10% in other populations.

## 1. Introduction

Cancer is a multifactorial disease associated with genetic and non-genetic risk factors [1]. HCSs are genetic diseases characterized by an increased risk of developing cancer caused by pathogenic germline variants that can be inherited in a mendelian fashion. HCSs are responsible for approximately 5–10% of all cancer cases [2,3]. To date, around 200 cancer susceptibility syndromes have been described; some of the most frequent include HBOC, Lynch syndrome, neurofibromatosis, tuberous sclerosis complex, familial adenomatous polyposis (FAP), familial malignant melanoma, hereditary retinoblastoma, familial Wilms tumor, type II multiple endocrine neoplasia, etc. [4]. To assess and diagnose individuals with a high risk of developing cancer, genetic counseling is essential in oncology centers [4,5].

Genetic counseling is an educational process that seeks to assist affected and/or at-risk individuals to better understand the nature of a genetic disorder, its transmission, and the options in management and family planning [6]. Dr. Sheldon Reed coined the genetic counseling term in 1947; this term came into use to teach other physicians about hereditary conditions. In the 1960s, medical genetics was recognized as a medical specialty, boosting genetic counseling; currently, 74 years later, it is internationally recognized as a vital part of medicine. In 2018, at least 28 countries had genetic counselors and/or medical geneticists as part of their medical services [7,8]. There is evidence of significant empowerment improvement in patients receiving cancer genetic counseling. A geneticist alleviates negative emotions in patients during genetic counseling [9].

An increasing demand for genetic services has led to the development of streamlined genetic counseling models [10]. Traditional genetic counseling models include a face-to-face pretest conversation with a geneticist, followed by a second in-person interview to review the results and related medical management recommendations, discuss psychological implications, and coordinate family cascade testing, as indicated [11,12]. Other approaches include telemedicine, implemented during the recent pandemic years, and group counseling sessions with positive results [10,13].

In the era of next-generation sequencing (NGS), genetic counselors explore ways to adapt counseling models to respond to the increase in patients who are eligible for genetic testing or have genetic test results that require genetic counseling [12]. NGS in germline DNA results could reveal HCS variants and mutations associated with other genetic diseases. Disclosing germline data could be clinically relevant and even lifesaving [14].

The World Health Organization (WHO), in its Human Genomics in Global Health Initiative, recommends a standard distribution of medical geneticists of 1 per 100,000 inhabitants [7,15]; this cannot be achieved in Latin-American countries. In Mexico, the level of knowledge of genetic disorders needs to be improved by physicians. There are around 300 certified medical geneticists (1 per 525,000 inhabitants), most of them in the three main cities (Mexico City, Guadalajara, and Monterrey). This, with other socioeconomic factors, explains the lack of access [15]. In Mexico, oncology centers must have at least one medical geneticist and an early detection program. The government does not widely support genetic testing because of the low demand and a lack of a specific program limited only to private centers [16]. The disparity between genetic counseling and testing can be seen in developing and developed countries with better health systems. In 2015, in the USA, the National Health Interview Survey (NHIS) reported that of a sample of 2,498,842 individuals with risk factors, only 378 reported genetic testing (0.015%) [17].

Pathogenic germline variants identification in cancer predisposition genes can impact clinical decisions affecting patient management, therapy, and surveillance [18]. This research aimed to describe the experience of implementing an HSC reference center, genetic counseling, and germline variants testing in an oncologic center in Monterrey, Mexico. Our center receives patients from the Nuevo León metropolitan area and the northeastern region of Mexico.

## 2. Materials and Methods

### 2.1. Patients’ Groups and Approval from the Ethics Committee

The study was conducted in the Centro Universitario Contra el Cáncer (CUCC) in agreement with the Declaration of Helsinki. The protocol was approved by the Institutional Ethics Committee of the University Hospital “Dr. José Eleuterio González” (registration number ON18-00015).

All patients were invited to participate in this research project, an interview was performed, and they signed an informed consent letter once they agreed to participate. Patients were recruited from the Instituto Mexicano del Seguro Social, Instituto de Seguridad y Servicios Sociales de los Trabajadores del Estado, Hospital Regional Materno Infantil, and Hospital Universitario “Dr. Jose Eleuterio González” as public institutions, and Oncare Clinical Center as the only private institution. Afterward, clinical and epidemiological information was collected, and blood samples were taken. A total of 3283 individuals referred to our center for genetic counseling between June 2016 and April 2022 were evaluated.

### 2.2. Algorithm Workflow

**Figure 1:**
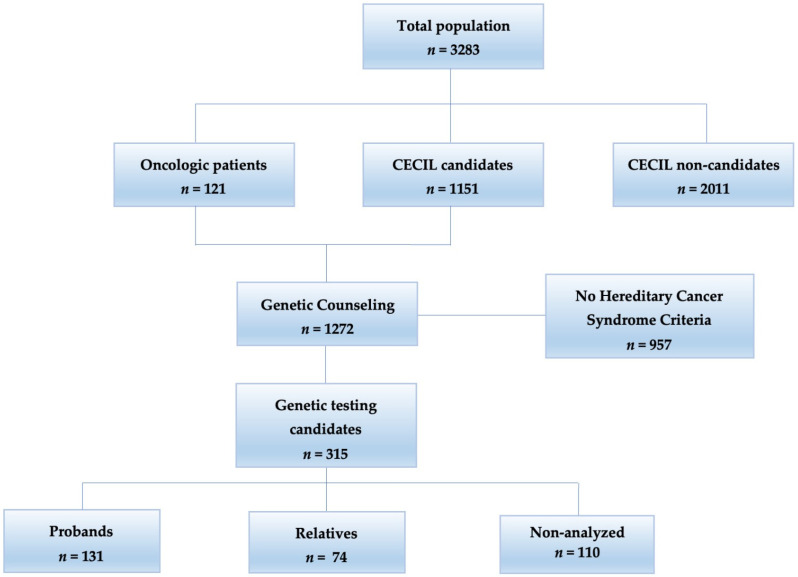
Flowchart of genetic counseling: of the total population (*n* = 3283), 315 patients were candidates for genetic testing.

The CUCC Early Cancer Detection Clinic (CECIL) implemented a cancer prevention model. The open population could enroll in a cancer prevention program, including screening for the most common cancers, nutritional counseling, and psychiatric evaluation. All patients were filtered by relative risk using a survey form with 16 questions (File S1). Trained medical assistants applied the survey prior to consultation; those patients who answered “yes” to at least one question were considered patients suspected of hereditary cancer. The patients were invited to CECIL, where a medical geneticist evaluated the probability of having a pathogenic germline variant. All patients received genetic counseling according to the American College of Medical Genetics (ACMG) [19] and National Comprehensive Cancer Network (NCCN) guidelines [17]. After consultation, genetic testing was offered, and in patients with germline variants, genetic test evaluation was offered for their relatives (Figure 1).

### 2.3. Proband Patients NGS Study

Saliva or peripheral blood samples were taken from proband patients. Different arrays of genetic testing were performed by external services, including gene panels from 30 to 84 genes and exome sequencing, depending on the study’s sponsor. Three patients had only *BRCA1/2* because they underwent direct-to-customer testing. Patients who paid for their research usually selected the 84 gene panel (Invitae Multi-Cancer Panel) from ^©^Invitae Corporation (San Francisco, CA, USA) because of cost–benefit, considering that other panels with fewer analyzed genes were equally expensive. Other patients that were partially sponsored by Foundations or had a donation were tested with the 30 gene panel (Onco Life test^®^) from Life in Genomics^®^ (Ciudad de Mexico, Mexico). Finally, some patients had the whole exome analyzed with an Illumina platform (San Diego, CA, USA).

### 2.4. Genetic Counseling

All patients had genetic counseling regardless of whether they had a genetic condition; if the patient had a familial or personal history of cancer, they were classified as sporadic, familial, or hereditary cancer cases; those who could afford the testing were counseled according to their result. All the patients receive their test result reports face to face from the geneticist, so we ensure that all participants accurately understand their test results. Those who tested positive for genetic tests entered a strict cancer early detection and prevention program. We offered biochemical, imaging studies, and risk reduction surgeries in selected cases. In cases where the test resulted in an unknown significance variant (VUS), an additional clinical case evaluation was performed. Additionally, a periodic reevaluation of the variant and a prevention program was personalized if necessary. Finally, if the test was negative, no additional evaluation was needed. For those who could not have genetic testing, an empiric prevention program was implemented as if they were positive, with the restriction that no risk reduction surgeries were allowed.

### 2.5. Test for Relatives and DNA Extraction

Following the proband diagnosis, relatives at risk had genetic counseling, and the genetic test was offered; all patients were evaluated in the psycho-oncology consultation before the test. All relative’s testing was performed by in-house Sanger sequencing. Patients with a positive Sanger sequencing test entered the prevention and early detection program.

Peripheral blood samples (5 mL) from the proband patient and relatives were collected by venipuncture. Samples were centrifuged at 3500 rpm for 10 min at room temperature. Genomic DNA was isolated from leukocytes using the QIAamp DNA Blood Midi kit (QIAGEN, Hilden, Germany) following the manufacturer’s recommendations. DNA was quantified by measuring the optical density (OD) at 260 nm using the QIAxpert UV/Vis spectrophotometer (QIAGEN, Hilden, Germany). The ratio OD260/OD280 determined the purity of DNA; values between 1.8 and 2 were considered pure. Genomic DNA was stored at −80 °C until use.

### 2.6. DNA Sanger Sequencing for Relatives

All pathogenic, likely pathogenic variants, and VUS were confirmed by Sanger Sequencing (primer sequences and conditions available upon request). DNA from the proband was used as a positive control. Specific primers for germline variants were designed in the Oligo^®^ Primer Analysis Software v7.60 (Cascade, CO, USA) [20]. The PCR products were purified with Wizard^®^ SV Gel and PCR Clean-Up System from Promega (Fitchburg, WI, USA) following the manufacturer’s instructions. The PCR products were subsequently subjected to sequencing with BigDye terminator v1.1 cycle sequencing reagents, purified with a BigDye XTerminator™ (Waltham, MA, USA) purification kit according to the manufacturer’s recommendations, and analyzed on an ABI 3130 Genetic analyzer (Applied Biosystems, Foster City, CA, USA).

### 2.7. Bioinformatics Analysis and Variant Annotation

To analyze the sequences, we used Sequencing Analysis v5.2 and SeqScape v2.6 software (Applied Biosystems, Foster City, CA, USA). For the annotation and clinical significance of all identified variants, we used the standards and guidelines for the interpretation of sequence variants recommended by the ACMG Laboratory Quality Assurance Committee) and the Association for Molecular Pathology (AMP) [19,21]. Disease-specific information for variants was retrieved from ClinVar [22], and the Online Mendelian Inheritance in Man (OMIM) database [23]. Novel pathogenic variants were reported in the ClinVar database from NCBI. All variant information was continuously updated and upgraded in the central database.

## 3. Results

### 3.1. Patient Description

In a period of 6 years (from June 2016 to April 2022), the cancer prevention program at the CECIL recruited a total of 3283 patients: 2011 patients (61.25%) were classified as non-candidates, and 1272 (38.74%) as candidates for genetic counseling (121 oncologic patients referred from different oncologic centers, and 1151 open population self-referred patients), according to both filters (Figure 1).

All patients and open population candidates had a genetic evaluation (*n* = 1272). A total of 957 (75.23%) did not fulfill the criteria for testing. Among the 315 (24.76%) candidates for testing, 110 (34.92%) could not take the test because of economic limitations. There was only one patient that did not accept the test. Of a total of 205 genetic tested individuals, 131 (63.90%) were probands, and 74 (36.09%) were relatives (Figure 1).

Of the 205 tested individuals, 121 were cancer patients (78 from CECIL, 33 were referred from public health institutions and 10 from a private clinic). The cancer distribution in the population was as follows: 95 (78.51%) had breast cancer unilateral, bilateral, or in combination with another tumor, followed by ovarian cancer 10 (8.26%), colon 8 (6.61%), endometrial cancer 3 (2.47%), and 5 (4.13%) others (Table 1).

Of the 131 probands, 49 patients (37.4%) came from the oncology consultation, 33 (25.19%) from other public health centers, 14 (10.68%) from private oncology services, and 35 (26.71%) self-referred. The mean age of the probands was 41.7 years old, the median age was 40.3 years, and it has a distribution of 123 females and eight males.

All the relatives were evaluated at CECIL (*n* = 74). The mean age of the relatives was 37.6 years old, the median 34 years, with an allocation of 53 females and 21 males.

### 3.2. Germline Variants

To identify variants predisposing to cancer, we analyzed 131 probands. We determined that 82 patients (62.59%) had at least one germline variant associated with cancer predisposition syndromes, of which 52 (63.41%) presented a pathogenic or probably pathogenic variant, and 40 (48.78%) had at least one VUS. No variants were detected in the remaining 49 patients (37.40%). Among the 74 relatives, 38 (51.35%) were positive for at least one germline variant.

From the probands, a 30-gene panel made molecular diagnoses in 64 patients (48.85%), followed by an 84 gene panel in 52 (39.69%), exome sequencing in 12 (9.16%), and 2 gene panels in 2 (1.52%). Most relatives were evaluated using a specific house-made primer design for the germline variant using Sanger sequencing (98.6%). Only one relative was tested with the 84 gene panel.

Among the 205 probands and relatives that were analyzed, all variants were classified according to the ACMG guidelines [16], most of the variants we found matched the clinical diagnosis or were easy to interpret, but in cases such a VUS, additional efforts had to be made to reclassify them. All the variants found were heterozygous, so autosomal recessive syndromes were discarded. In probands, funding for the studies came from various sources: 66 (50.38%) could afford the total price, 47 (35.87%) had partial sponsors of foundations, 13 (9.92%) were paid by the Hospital as part of research protocols, and 5 (3.81%) were paid by private donations. Among relatives, 64 (86.48%) paid for their studies, and 10 (13.51%) were paid by CECIL.

Among the 131 probands, we found a novel variant in *APC* (del ex 5 c.422+1123_532-577delins423-1933_423-1687inv). Additionally, we found founder mutations in *BRCA1*: c.68_69delAG (p.Glu23Valfs*17), c.211A > G p.(Arg71Gly), c.5123C > A (p.Ala1708Glu), and deletion (ex 9–12) and one for *MUTYH* c.118G > A (p.Gly396Asp). The most frequent variants for breast cancer were in *BRCA1* (deletion (ex 9–12) and c.115T > A (p.Cys39Ser)) and *MUTYH* c.118G > A (p.Gly396Asp); these variants were found in four patients each. In colon cancer, there were two for *MLH1* c.1790_1791delins ATCTGGACC and c.676C > T (File S1).

### 3.3. HCS Frequency

**Figure 2:**
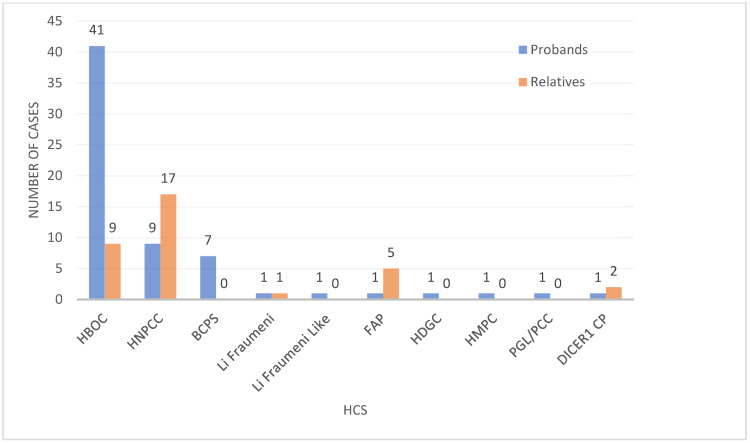
HCS frequency: Distribution of the variants in probands and relatives and their diagnosis. One proband was a double heterozygous for hereditary paraganglioma-pheochromocytoma (PGL/PCC) and *DICER1* cancer predisposition (*DICER1* CP) syndrome. FAP, HDGC: hereditary diffuse gastric cancer syndrome, HMPC: hereditary melanoma-pancreas cancer predisposition syndrome, PGL/PCC.

The most frequent clinically diagnosed syndromes in probands were HBOC with 41 cases (*BRCA1* in most cases), followed by eight cases of Lynch syndrome with *MLH1* as the primarily responsible gene, and seven cases of breast cancer predisposition syndrome. For the rest of the syndromes, we found one case of each (*n* = 7) (Figure 2). In the relatives’ group, there were 17 Lynch syndrome cases, followed by nine cases of HBOC, two cases of *DICER1* cancer predisposition syndrome, one case of Li Fraumeni syndrome, and four cases of FAP. In 19 patients with high clinical suspicion of hereditary cancer due to clinical and familial history of cancer, we found seven with VUS, probably benign and benign variants, and 12 without any variant.

## 4. Discussion

The WHO Human Genetics Program has recognized the need for genetic services worldwide. It achieves various public health goals such as reducing health disparities and preventing, diagnosing, and treating genetic diseases [24,25]. In Latin America, the lack of access to medical genetics services poses a tremendous challenge to fulfilling the objectives of the WHO [15,26]. In Mexico, public institutions continue to be the leading providers of genetic services for most of the population, mainly by enrolling patients in research protocols. Access to medical genetics is difficult because they are primarily available in the three major cities in the country [15,26,27].

This report found some interesting results, including a novel variant in *APC* (del ex5 c.422+1123_532-577delins423-1933_423-1687inv) in a family with an atypical clinical presentation that led to the creation of an in-house process of detection for the whole family [28]. Additionally, there were founder mutations in *BRCA1*, c.68_69delAG (p.Glu23Valfs*17) in one patient known in the Ashkenazi Jew population, and two cases of *BRCA1* c.211A > G p.(Arg71Gly) reported in the Polish and Lithuanian populations. There were two cases of *BRCA1* c.5123C > A (p.Ala1708Glu) reported in the Spanish population.

We also found the Mexican founder mutation in *BRCA1* del (ex 9–12) in four cases. In other studies, in Hispanic patients, the frequency of this pathogenic variant was between 43% and 62% in *BRCA* pathogenic variants carriers (mainly Mexican American and Central and Southern Mexican populations). On the other hand, a study in the northeastern Mexico region found a frequency of 21.4% [29,30,31]. Our results showed a frequency of 13.3%, which is lower than the other studies, but near the results of the northern population. The Mexican population is mainly mestizo from Amerindian–Spanish admixture (93%). There is significant differentiation among mestizos between the northwest when compared with the central and southeast regions of Mexico [32]. In Mexico, around 55% of ancestral genes are Indian, 40% Caucasian, and 5% African American. In some coastal areas, however, as much as 40% African American ancestry is present, with a more Caucasian component in the northern regions [33,34,35].

Additionally, of the *BRCA* pathogenic variants, seven patients had a clinical diagnosis of HCS due to an extensive personal and familial history of related cancers. Still, they presented variants classified as non-pathogenic in other populations (Table S1). Among these variants, we suggest that the likely benign variant *BRCA1* c.3113A > C (ClinVar report last evaluated in 2013 [36]) is probably a pathogenic variant in the Mexican population. In our series, this variant was detected in two non-related families with personal histories of multiple cancer and multiple familial accounts of related tumors. Based on this data, testing was offered to other family members; unfortunately, most of the individuals died of cancer at the time of the analysis. We detected this variant in two female relatives: a 35-year-old female with breast cancer and a 27-year-old female in follow-up as a previvor patient. Other relatives are deciding if they want to be tested. Reclassifying this variant requires additional research and clinical confirmation in more individuals.

Due to the mestizo Amerindian–Spanish admixture of the Mexican population, variant interpretation is complex. Due to miscegenation in the Mexican population, some variants considered non-pathogenic for other populations should be reclassified for our population, according to clinical findings.

As expected by the global frequency, other non-*BRCA* genes related to hereditary breast cancer variants were found (*PALB2* and *CHEK2*) [37,38]. Additionally, we found two cases with succinate dehydrogenase complex flavoprotein subunit A (*SDHA*) pathogenic variants; this gene is related to hereditary paraganglioma-pheochromocytoma syndrome, but has been found in two breast cancer cases [39]. Therefore, using multigene panels allows us to detect unknown, low-frequency genes or misclassified variants in the Latino population. Generating robust data on our Mexican population will help us to modify or create new guidelines for the follow-up of these patients.

At the time of publication of this paper, we have detected premalignant lesions in four patients, three diagnosed as HNPCC (two with colonic premalignant lesions, and one an in situ endometrial cancer). The other patient (an 11-year-old girl) has FAP and glycogenosis, and we found three premalignant lesions in the colon. All patients were treated, the lesions were removed, and they continued their prevention screening program free of cancer. Of the HNPCC patients, one could not afford the genetic testing, but had the empirical follow-up.

Our cancer prevention model includes the evaluation of nutritional status, mental health, and a screening study of the most frequent types of cancer according to the patient’s characteristics, instead of many prevention models that only focus on physiological, environmental, and genetic risk factors [40,41,42]. We propose that this model can significantly decrease healthcare costs, public health, and cancer control prevalence [40,42,43,44].

Genetic counseling allows patients and their family members to receive education about the benefits, limitations, and risks of genetic testing, as well as the early detection and prevention strategies, to make an informed decision [45]. We have a high pathogenic variant detection rate (40%) in probands; other studies with different methodologies and models detect 10–20% in selected populations. Our purpose was to provide concise coverage of the significant HCS.

In addition to the selected population from the oncologic center, our model included an open population of self-referred patients filtered by a survey and general practitioner before the genetic consultation. From the 35 self-referred probands, we identified 16 patients (45.71%) with pathogenic variants. This group of self-referred probands is frequently an overlooked population, and now, thanks to the CECIL model, they have a diagnosis and genetic counseling.

The CECIL model includes educational sessions for patients, relatives, medical staff, and the general population, including cancer prevention strategies and broad genetic counseling talks. We also include psychoeducational sessions. We decided to continue with the traditional face-to-face approach as our primary strategy because of the economic limitations of the patients who attended CECIL. Telemedicine is also available as an option for foreign patients.

As seen in other genetic counseling strategies, part of our model includes educational sessions for patients, relatives, medical staff, and the general population, including cancer prevention strategies, psychoeducational sessions, and broad genetic counseling to create awareness, in combination with the traditional face to face approach, which in our case resulted to be a successful strategy for our model.

Currently, none of the public health services in Mexico offer genetic testing for hereditary cancer as part of their coverage camp [46]. In 2022, based on the experience of the CECIL program, the Nuevo Leon government approved a sponsor program for free genetic testing in breast cancer, taking the first step in accessing molecular diagnosis in Mexico [47].

## 5. Conclusions

Genetic counseling in HCS is still a global challenge; the access to affordable testing in developed countries gap is shortening thanks to cheaper technologies and the involvement of public and private health initiatives. With the CECIL model, we could implement a hereditary cancer database, discover novel variants, and suggest reclassifying the pathogenicity of variants.

The advantages of establishing a unified genetic counseling strategy such as the CECIL model include: establishing a database of HCS families and variants, more detailed analysis of epidemiological data, the establishment of the frequency of the variants in the studied population, discovery of novel pathogenic variants, and variants linked to specific populations. It leads to better outcomes and a more supervised follow-up for our patients and their relatives. Additionally, an integral prevention program ensures that all patients receive adequate medical and psychological assistance as detailed by international guidelines.

The detection of a pathogenic variant in northeast Mexico leads to establishing prevention and early detection programs. The success of the CECIL model can lead to the formation of other prevention oncology centers. In our case, it was the beginning of a state program for all public health institutions to achieve broad coverage. The main goal is to extend the program nationally.

## Figures and Tables

**Table 1:** Cancer distribution: A total of 121 cancer patients were distributed by frequency.

Cancer Distribution	*n* (%)
Breast (total) Breast (Unilateral) Breast (Bilateral) Breast (Bilateral) & Pancreas Breast & Pancreas Breast and Ovarian	95 (78.51) 82 (86.31) 8 (8.42) 1 (1.05) 1 (1.95) 3 (3.15)
Ovarian (total) Ovarian (unilateral) Ovarian, Kidney and Pseudomyxoma	10 (8.26) 9 (90) 1 (10)
Colon	8 (6.61)
Melanoma, Breast, and Cervical	1 (0.82)
Endometrial	3 (2.47)
Sarcoma (total) Sarcoma Sarcoma and Thyroid	2 (1.65) 1 (50) 1 (50)
Medulloblastoma and Kidney	1 (0.82)
Hemangioendothelioma	1 (0.82)

## Data Availability

The novel APC pathogenic variant was submitted in the ClinVar database by the following variation ID 988590 (https://www.ncbi.nlm.nih.gov/clinvar/variation/988590/, accessed on 2 January 2023) and in the next research article doi:10.3390/diagnostics11030411, accessed on 15 November 2022).

## Supplementary Materials

The following supporting information can be downloaded at: https://www.mdpi.com/article/10.3390/genes14020341/s1, File S1: Genetic reference survey; Table S1: Genetic testing database.
