# Supplementary material for: Identification of Germline Variants in Patients with Hereditary Cancer Syndromes in Northeast Mexico
Source: Genes (Basel). 2023 Jan 28;14(2):341. doi: 10.3390/genes14020341 (PMC9957276; doi:10.3390/genes14020341)
Supplement: Supplementary file 1 [file genes-14-00341-s001.zip › Sumplementary File S1.pdf]

HOSPITAL UNIVERSITARIO JOSÉ ELEUTERIO GONZALEZ
CENTRO UNIVERSITARIO CONTRA EL CANCER
SERVICIO DE ONCOLOGÍA

GENETIC REFERENCE SURVEY

Date: ____________________ Register #: ____________________________________

Name: ____

Cancer type and clinical staging: ____

	yes/no
• Two or more cancer cases in one family side	[ ]
• More than one relative with cancer in two generations (on one family side)	[ ]
• Early onset (cancer under 40 years)	[ ]
• Multiple primary tumors (≥2) in the patient	[ ]
• Bilateral cancer (Ex: Breast, ovarian) or Multifocal (Ex: ≥2 tumors in the same organ)	[ ]
• Rare tumors	[ ]
• Cancer patient with a birth defect (Short or tall stature, segmental overgrowth, macro or microcephaly, cleft lip-palate etc.)	[ ]
• Ovarian cancer	[ ]
• Triple negative breast cancer	[ ]
• Familial Adenomatous Polyposis	[ ]
• Multiglandular disease (Ex. Thyroid, Parathyroid, adrenal gland)	[ ]
• Primary Hyperthyroidism	[ ]
• Medullary Thyroid cancer	[ ]
• Hemangioblastoma (Benign vascular lesion)	[ ]
• Clear cell renal cancer	[ ]
• Diagnosed with hereditary cancer	[ ]

IN CASE OF ONE OR MORE “YES”, PLEASE SEND THE PATIENT TO GENETIC COUNSELING
